# Intricate Macrophage-Colorectal Cancer Cell Communication in Response to Radiation

**DOI:** 10.1371/journal.pone.0160891

**Published:** 2016-08-11

**Authors:** Ana T. Pinto, Marta L. Pinto, Sérgia Velho, Marta T. Pinto, Ana P. Cardoso, Rita Figueira, Armanda Monteiro, Margarida Marques, Raquel Seruca, Mário A. Barbosa, Marc Mareel, Maria J. Oliveira, Sónia Rocha

**Affiliations:** 1 i3s-Instituto de Investigação e Inovação em Saúde, Universidade do Porto, Porto, Portugal; 2 INEB-Institute of Biomedical Engineering, University of Porto, Porto, Portugal; 3 FEUP-Faculty of Engineering, University of Porto, Porto, Portugal; 4 ICBAS-Institute of Biomedical Sciences Abel Salazar, University of Porto, Porto, Portugal; 5 IPATIMUP-Institute of Molecular Pathology and Immunology of the University of Porto, Porto, Portugal; 6 Radiotherapy Service, Centro Hospitalar S. João, EPE, Porto, Portugal; 7 Department of Pathology and Oncology, Faculty of Medicine, University of Porto, Porto, Portugal; 8 Department of Radiation Oncology and Experimental Cancer Research, Ghent University Hospital, Ghent, Belgium; 9 Centre for Gene Regulation and Expression, College of Life Sciences, University of Dundee, Dundee, United Kingdom; Technische Universitat Dresden, GERMANY

## Abstract

Both cancer and tumour-associated host cells are exposed to ionizing radiation when a tumour is subjected to radiotherapy. Macrophages frequently constitute the most abundant tumour-associated immune population, playing a role in tumour progression and response to therapy. The present work aimed to evaluate the importance of macrophage-cancer cell communication in the cellular response to radiation. To address this question, we established monocultures and indirect co-cultures of human monocyte-derived macrophages with RKO or SW1463 colorectal cancer cells, which exhibit higher and lower radiation sensitivity, respectively. Mono- and co-cultures were then irradiated with 5 cumulative doses, in a similar fractionated scheme to that used during cancer patients’ treatment (2 Gy/fraction/day). Our results demonstrated that macrophages sensitize RKO to radiation-induced apoptosis, while protecting SW1463 cells. Additionally, the co-culture with macrophages increased the mRNA expression of metabolism- and survival-related genes more in SW1463 than in RKO. The presence of macrophages also upregulated glucose transporter 1 expression in irradiated SW1463, but not in RKO cells. In addition, the influence of cancer cells on the expression of pro- and anti-inflammatory macrophage markers, upon radiation exposure, was also evaluated. In the presence of RKO or SW1463, irradiated macrophages exhibit higher levels of pro-inflammatory *TNF*, *IL6*, *CCL2* and *CCR7*, and of anti-inflammatory *CCL18*. However, RKO cells induce an increase of macrophage pro-inflammatory *IL1B*, while SW1463 cells promote higher pro-inflammatory *CXCL8* and *CD80*, and also anti-inflammatory *VCAN* and *IL10* levels. Thus, our data demonstrated that macrophages and cancer cells mutually influence their response to radiation. Notably, conditioned medium from irradiated co-cultures increased non-irradiated RKO cell migration and invasion and did not impact on angiogenesis in a chicken embryo chorioallantoic membrane assay. Overall, the establishment of primary human macrophage-cancer cell co-cultures revealed an intricate cell communication in response to ionizing radiation, which should be considered when developing therapies adjuvant to radiotherapy.

## Introduction

Tumours are complex ecosystems involving much more than solely cancer cells. They are characterized by a dynamic tumour microenvironment supported by extracellular matrix components and several tumour-associated cells, which altogether modulate cancer cell activities, dictating the success of tumour progression [1, 2]. Amongst tumour-associated cells, macrophages are particularly relevant, as they constitute, in many solid tumours, the most abundant immune population, and are known as obligate partners for cancer cell migration, invasion and metastasis [3, 4].

Macrophages not only contribute to tumour progression, as they may also modulate tumour response to therapy [5, 6], particularly to radiotherapy, one of the most common anti-cancer treatments, employed in approximately 50% of all cancer patients at some point of their treatment [7]. Radiotherapy is typically delivered as a multi-fractionated rather than single-dose regimen, involving daily doses of 2 Gy (5 fractions/week), during several weeks of treatment [8]. Notably, the anti-tumour effects of macrophage depletion seem to support their role in promoting tumorigenesis [9, 10]. In fact, in animal models, depletion of tumour-associated macrophages, either locally or systemically, prior to radiotherapy, decreases tumour regrowth, favouring the anti-tumour effects of ionizing radiation [10]. Consequently, co-implantation of tumour cells with bone marrow–derived macrophages increases tumour radioresistance [10], although macrophages are also able to radiosensitize tumour cells, for instance through the induction of NO synthesis [9].

In disease as well as in homeostasis, macrophages exhibit a functional phenotype that may vary between two extremes of a continuous spectrum of activation: pro-inflammatory and anti-inflammatory macrophages, commonly known as M1- and M2-like macrophages, respectively [11]. Pro-inflammatory macrophages are generally characterized by the production of high levels of pro-inflammatory mediators, such as TNF-α, IL1-β, IL-6 or IL-12 and are associated with bacterial clearance and tumour cytotoxicity, being considered tumour suppressors [11]. In contrast, anti-inflammatory macrophages are high producers of anti-inflammatory mediators, such as IL-10 or TGF-β, and are mainly involved in extracellular matrix remodelling and immune suppression, being considered tumour promoters [11]. In tumours, macrophages frequently acquire an anti-inflammatory profile [12] and their modulation towards a pro-inflammatory phenotype has been pointed as another possible strategy to modulate tumour cell response to therapy [6, 13].

Although macrophages may play a role in tumour cell radioresistance, this may also be intrinsically determined, namely by *p53* mutations [14, 15] and chromosomal instability in tumour cells [16]. Additionally, alterations in DNA repair efficiency [17], upregulation of the pro-survival protein Bcl-xL [18], enhanced aerobic glycolysis [14], and altered mitochondrial function [19] may also contribute to acquired resistance to radiation-induced apoptosis. Overall, cancer cell response to radiation has been intensively investigated and it is now well-characterized [20]. Nonetheless, only more recently, attention has been paid to the effect of radiation on tumour-associated host cells, as they were found to play a role in radiotherapy outcome [21, 22]. We have recently provided new insights into this field, by exploring the effect of clinically relevant ionizing radiation doses on macrophage function and survival [23].

To design effective therapies to radiosensitize cancer cells in the presence of macrophages, it is crucial to understand how macrophage and cancer cell communication affects the response to radiation. To address this issue, we established indirect macrophage-cancer cell co-cultures and exposed the whole system to cumulative ionizing radiation doses, in a fractionated scheme similar to the one used for cancer patients’ treatment (2 Gy/fraction/day). We selected two colorectal cancer cell lines, RKO and SW1463, known to exhibit, respectively, a radiation sensitive [24] and a radiation resistant profile [25], and co-cultured them with human monocyte-derived macrophages. This *in vitro* approach constitutes the basis of the present study and allowed us to characterize two important hallmarks of cancer cells, the resistance to cell death and the deregulation of cellular energetics, and to profile the macrophage activation state upon co-culture irradiation. Additionally, the role of irradiated co-culture conditioned medium on other hallmarks of cancer, namely cell invasion, migration and angiogenesis was also addressed.

## Material and Methods

### Ethics statement

In the present study, human monocytes were isolated from buffy coats of healthy blood donors, obtained through a collaboration protocol with Centro Hospitalar São João (CHSJ). This was approved by CHSJ Ethics Committee for Health (References 259 and 260/11), in agreement with the Helsinki declaration. A written informed consent was obtained from all subjects before blood donation. Buffy coats were provided anonymised, and their identification was only accessible to hospital staff.

### Human monocyte isolation and macrophage differentiation

Human monocytes were isolated from healthy blood donors as previously described, using the RosetteSep monocyte-enrichment kit (StemCell) [23]. Following this negative separation procedure, over 85% of isolated monocytes were found to be CD14-positive [26]. For monocyte-macrophage differentiation, 1.2x10^6^ cells/9.6 cm^2^ were cultured in RPMI1640 medium (with GlutaMax) (Invitrogen, Merelbeke, Belgium) supplemented with 10% FBS (Lonza, Basel, Switzerland), 100 U/mL penicillin and 100 μg/mL streptomycin (Invitrogen), in the presence of 50 ng/mL of macrophage colony-stimulating factor (M-CSF) (ImmunoTools, Friesoythe, Germany). After 7 days, cell culture medium was replaced without M-CSF renewal.

### Cancer cells

Human RKO colon cancer cells were purchased from ATCC, while human SW1463 rectal cancer cells were kindly provided by Prof Kevin M. Haigis (Molecular Pathology Unit, Center for Cancer Research and Center for Systems Biology, Massachusetts General Hospital, USA). Cell DNA was analyzed with POWERPLEX 16 HS kit (Promega, Madison, WI, USA) and cell lines were tested and authenticated by autosomal STR DNA profiling at IPATIMUP Diagnostics, a laboratory accredited by the College of American Pathologists and with a Quality Management System certified in accordance with NP EN ISO 9001:2008 (IPATIMUP Diagnostics, Porto, Portugal). Both cell lines were cultured in RPMI1640 medium (with GlutaMax) (Invitrogen) supplemented with 10% FBS (Lonza), 100 U/mL penicillin and 100 μg/mL streptomycin (Invitrogen), at 37°C and 5% CO_2_.

### Establishment of macrophage-cancer cell co-cultures

**Fig 1:**
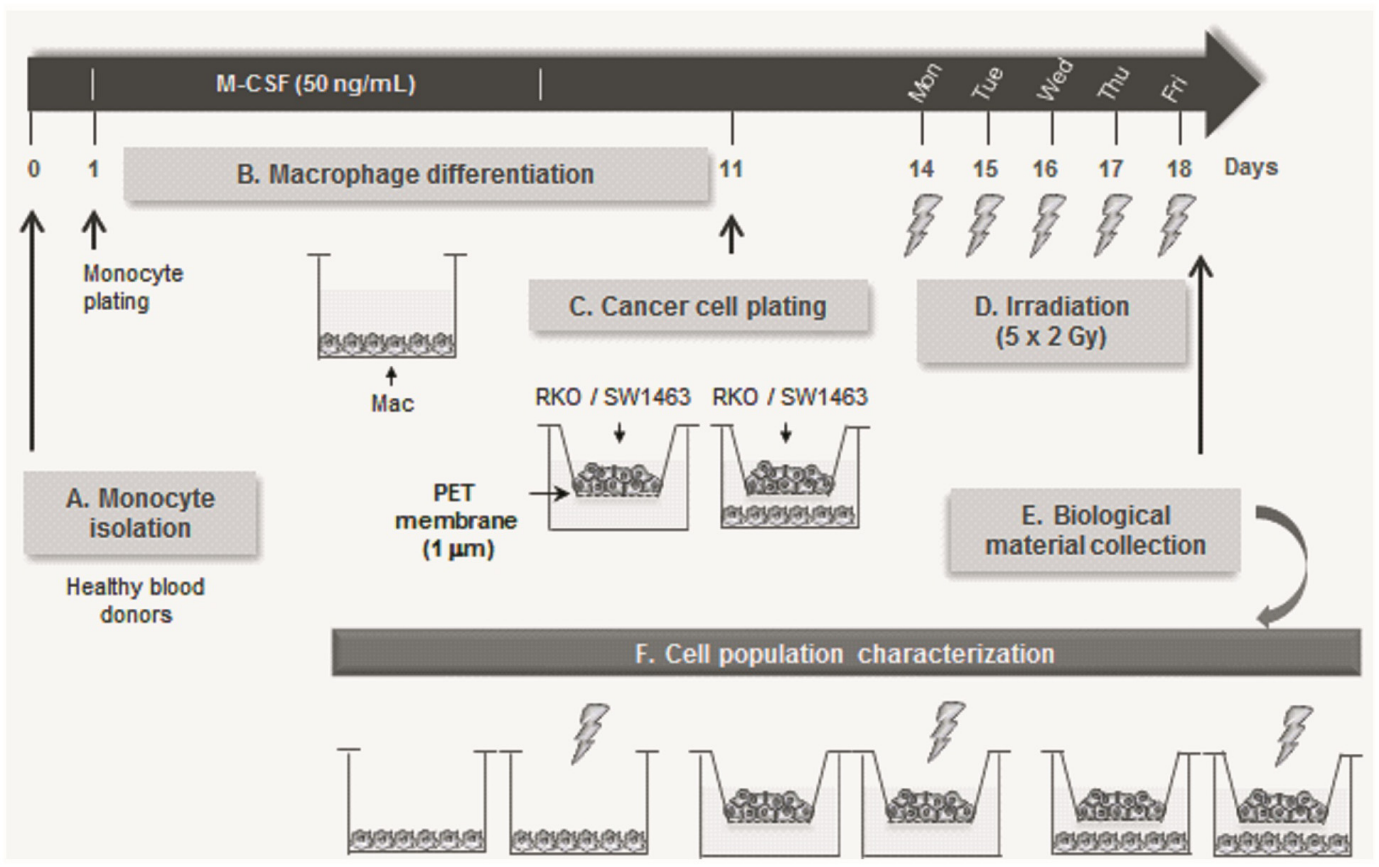
Schematic overview of the methodology used in this work. Monocytes were isolated from the peripheral blood of healthy blood donors, and cultured, for seven days, with M-CSF to allow their differentiation into macrophages. On day 11, RKO or SW1463 cancer cells were cultured in transwell inserts of 1 μm pore size, on top of macrophages, and the whole set was then irradiated with 2 Gy/fraction/day, for 5 days. Conditioned medium (CM) of irradiated co-cultures, as well as protein and RNA from individual cell populations, were collected 6 h after the last ionizing radiation dose, and compared with the respective controls.

Eleven days after monocyte isolation, RKO (12.6x10^3^ cells/well) or SW1463 (12.8–16.0x10^4^ cells/well) cancer cells were plated in 6 well-plate permeable transwell inserts (Corning, Cat. No. 353102, New York, USA), and placed on top of macrophages (Fig 1). The permeable PET membrane of 1.0 μm pore size avoided cancer cells to cross from the top to the lower compartment, where macrophages were previously differentiated, allowing however the exchange of soluble factors between both populations. Co-cultures were maintained in RPMI1640 medium (with GlutaMax) (Invitrogen) supplemented with 10% FBS (Lonza), 100 U/mL penicillin and 100 μg/mL streptomycin (Invitrogen) for 3 days before irradiation. Due to limited availability of human primary macrophage material, RKO and SW1463 cells were co-cultured with a different set of macrophages, derived from 4 distinct blood donors, in two independent experiments per each cell line. For control purposes, macrophage and cancer cell monocultures were also prepared.

### Ionizing radiation exposure

The dosimetry plan was established, as previously reported [23]. Cell culture medium was renewed before the first irradiation and mono- or co-cultures were exposed to cumulative ionizing radiation doses (2 Gy/fraction/day), for 5 days (5 x 2 Gy), totalizing 10 Gy. Photon beam was produced by a PRIMUS (Siemens, Malvern, PA, USA) linear particle accelerator, used for human radiotherapy, operated at 18 MV at the Radiotherapy Service of CHSJ. To avoid differences between non-irradiated and irradiated cells, caused by medium agitation during transport to/from the Radiotherapy Service, control cells were also transported, but were not exposed to radiation.

### Western Blot analysis

Total protein was extracted 6 h after cumulative ionizing radiation doses (5 x 2 Gy). Lysis buffer A [20 mM Tris-HCl (pH 7.5–8), 600 mM NaCl, 1% Igepal], or RIPA [50 mM Tris-HCl (pH 7.5), 150 mM NaCl, 2 mM EDTA and 1% Igepal] were supplemented with a cocktail of proteases and phosphatases inhibitors: phenylmethanesulfonylfluoride 1 mM, sodium metavanadate 3 mM, sodium fluoride 20 mM, sodium pyrophosphate tetrabasic 25 mM (Applichem), aprotinin 10 mg/ml and leupeptin 10 mg/ml (Sigma-Aldrich). Proteins extracted with Laemmli buffer 1x [3% glycerol, 5% β-mercaptoethanol, 2% SDS, 0.1% blue bromophenol in 1M Tris-HCl pH 6.8] were sonicated to shear DNA. Protein concentration was determined with Protein Assay Dye Reagent Concentrate (BioRad). Before loading into 10–15% SDS-polyacrylamide gels, proteins were diluted in Laemmli buffer containing β-mercaptoethanol (BioRad) and denatured at 95°C for 5 min. Primary antibodies against phosphorylated H2AX total (Ser139, ɣH2AX) (clone JBW301, dilution 1:500, Millipore) and cleaved PARP (clone D64E10), total and cleaved caspase-3, GLUT1, phospho-Chk2 (Thr 387) and Chk2 (dilution 1:1000, Cell Signalling) were used. Antibody against β-actin (dilution 1:10000, clone 8H10D10, Cell Signalling) was used to normalize protein expression. Goat anti-rabbit or horse anti-mouse-Horseradish peroxidase (HRP)-conjugated secondary antibodies (dilution 1:2000, Cell Signalling) were used, followed by ECL- detection (Thermo Fisher Scientific).

### RNA extraction, cDNA preparation and quantitative PCR analysis

Total RNA was extracted 6 h after irradiation (5 x 2 Gy), using TriPure Isolation Reagent (Roche), according to manufacturer´s instructions. RNA was converted to cDNA as previously described [23]. To evaluate mRNA expression levels, quantitative PCR using Brilliant II SYBR green kit (Stratagene/Agilent Technologies) and MX3005P qPCR platform (Stratagene/Agilent) was performed. The following primers were used for RT-qPCR: CXCL8, F:5′-CCAGGAAGAAACCACCGGA-3′, R:5′-GAAATCAGGAAGGCTGCCAAG-3′; IL1B, F:5′-GGCAGGGAACCAGCATC-3′, R:5′-CCGACCACCACTACAGCAA-3’; MCL1, F:5´-CAAGCAGAAGTGGGTTCAGGAT-3’, 5’-TCTTCGGAGTTTGGGTTTGC-3’; LDHA, F:5’-GGAGATCCATCATCTCTCCC-3´, R:5’-GGCCTGTGCCATCAGTATCT-3’ (Invitrogen); BCL2L1, F:5’-CTGCTGCATTGTTCCCATAG-3´, R:5’-TTCAGTGACCTGACATCCCA-3´; SLC2A1, F:5´-CGGGCCAAGAGTGTGCTAAA-3’, R:3’-TGACGATACCGGAGCCAATG-5’(Genomic Oligo); CCL5 F:5’-GTCGTCTTTGTCACCCGAAAG-3’, R:5’-TCCCGAACCCATTTCTTCTCT-3’. Primer sets for *ACTB* (used as a housekeeping gene) and *CCL2* were obtained from Qiagen, while probes for CD80, CCR7, TNF, IL6, CD163, IL10, CCL18, CSF1 and VCAN were from Applied Biosystems.

### Functional assays

To study the effect of conditioned medium (CM) from irradiated co-culture on the functional activity of non-irradiated cells, RKO or SW1463 were stimulated with CM from co-cultures collected 6 h after irradiation (5 x 2 Gy). For proper comparison, RKO cells were stimulated with CM from irradiated or non-irradiated macrophage-RKO co-cultures, while SW1463 were exposed to CM from irradiated or non-irradiated macrophage-SW1463 co-cultures and cancer cell migration and invasion were then evaluated. The angiogenic potential of CM from irradiated or non-irradiated co-cultures was directly evaluated using an *in vivo* model, without cancer cell inoculation.

#### Matrigel invasion assays

Non-irradiated RKO or SW1463 cells (5x10^4^) were seeded on the upper compartment of Matrigel-coated inserts with 8-μm pore size (BD Biosciences, Madrid, Spain) and stimulated with CM from irradiated or non-irradiated co-cultures for 24 h, at 37°C and 5% CO_2_. The porous membranes were then washed, fixed in 4% paraformaldehyde, mounted in Vectashield+4’,6-diamidino-2-phenylindole (DAPI, Vector Laboratories, Burlingame, CA, USA) for nuclei staining, and the number of invasive cells was counted on a fluorescence light microscope (Leica).

#### Migration assay

Non-irradiated RKO or SW1463 cancer cells were plated in individual culture-inserts (Ibidi, Cat. No. 80209, Munich, Germany), appropriated for 2D migration assays, and maintained at 37°C and 5% CO_2_ until confluence. These culture inserts were composed by two chambers separated by a biocompatible silicone material, which after removal allowed cells from each edge to migrate towards the centre of the gap. So, after barrier removal, confluent cancer cell monolayers were washed with PBS, to remove non-adherent cells, and stimulated with CM from irradiated or non-irradiated co-cultures. Stimulated cells were maintained at 37°C and 5% CO_2_ for 48 h. Cell migration was daily followed with photos acquired with the digital Camera EOS 1000D (Cannon) connected to a brightfield microscope (Zeiss).

#### Chick embryo chorioallantoic membrane (CAM) angiogenesis assay

Before performing the assay, CM from irradiated and non-irradiated co-cultures were concentrated around 10 times in a Savant SpeedVac Concentrator under vacuum (Thermo Scientific, Massachusetts, EUA). The chick embryo CAM model was used to evaluate the angiogenic potential of both CM, as previously described [23]. On embryonic development day (EDD)10, concentrated CM from irradiated or non-irradiated co-cultures were inoculated on top of the same CAM into two independent 3 mm silicone rings, under sterile conditions. Eggs were re-sealed and returned to the incubator for additional 72 h. On EDD13, rings were removed, the CAM was excised from embryos and photographed *ex-ovo* under a stereoscope, using a 20x magnification (Olympus, SZX16 coupled with a DP71 camera). The number of new vessels (< 20 μm diameter) growing radially towards the inoculation area was counted in a blind fashion manner. Eggs from two different batches were inoculated with CM from macrophage-RKO and macrophage-SW1463 co-cultures, obtained from 4 independent experiments (*n* = 15/condition).

### Statistical analysis

All graphs and statistical analysis were performed using GraphPad Prism Software v5 (GraphPad-trial version). As the recommended normality test—D’Agostino and Pearson required *n* ≥ 8 and the present study only involved comparisons with a maximum of *n* = 4, it was not possible to analyse data for Gaussian distribution. Therefore, *t*-test (paired, non-paired or one sample *t*-test) was used to compare data. Statistical significance was achieved when *P* < 0.05. *P < 0.05, **P < 0.01, *** *P* < 0.001, **** *P* < 0.0001.

## Results

To address the proposed goals, an indirect co-culture system was established, which involved the crosstalk of human monocyte-derived macrophages with two colorectal cancer cell lines, RKO or SW1463, known to exhibit high and low sensitivity to radiation, respectively [25, 24]. Monocultures and co-cultures were then subjected to cumulative ionizing radiation doses for 5 days, using a fractionated scheme similar to the one employed during cancer patients’ treatment (2 Gy/fraction/day) (Fig 1).

### The presence of macrophages reduces radiation-induced apoptosis in SW1463, but not in RKO cells

**Fig 2:**
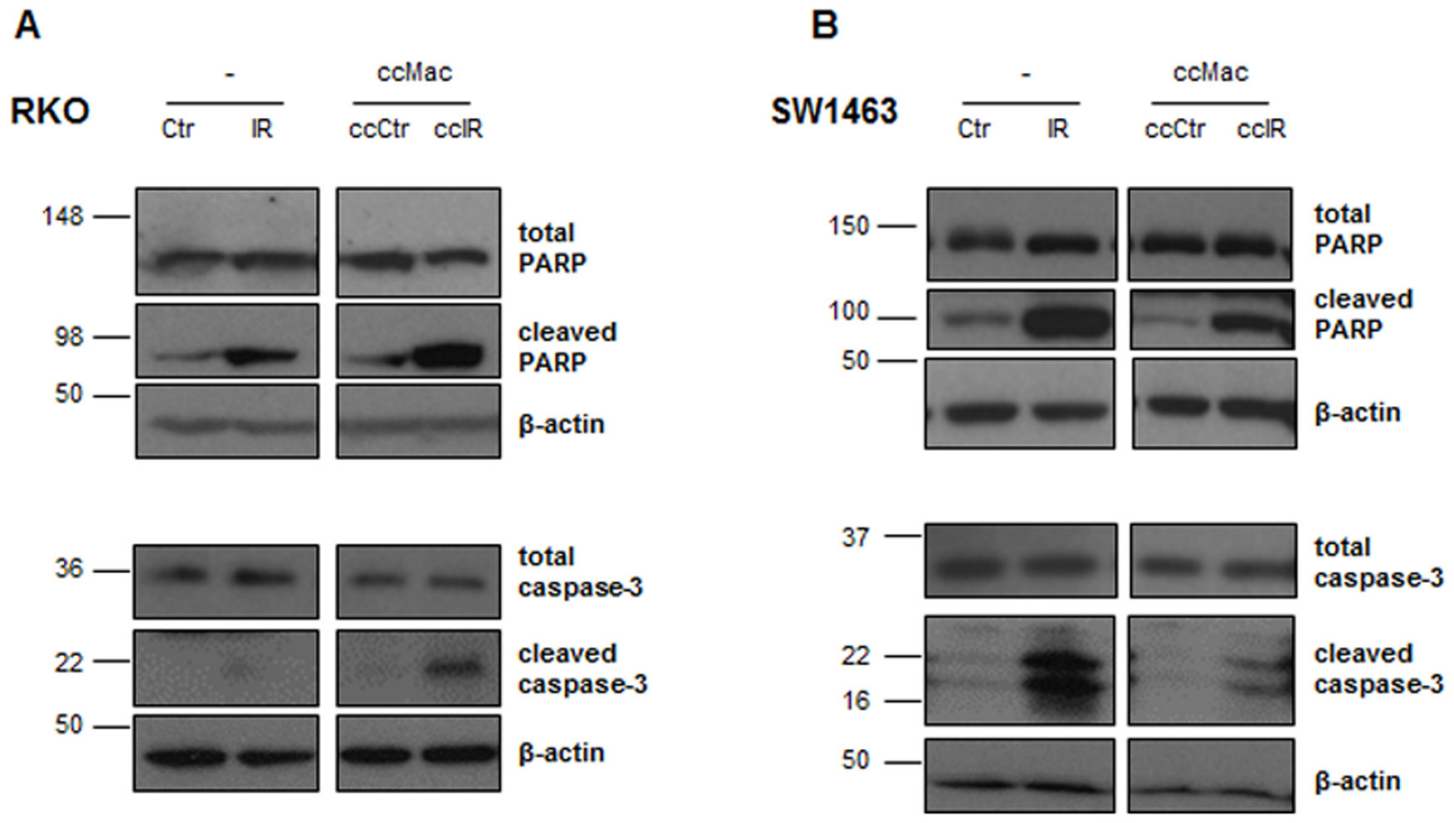
Expression of total and cleaved forms of PARP and caspase-3 in RKO and SW1463 cancer cells. **A)** RKO and **B)** SW1463 cancer cells were cultured alone (-) or in the presence of macrophages (ccMac), with (IR or ccIR, 5 x 2 Gy) or without (Ctr or ccCtr) radiation exposure. Expression levels of total and cleaved forms of PARP and caspase-3 were evaluated 6 h after irradiation by western blot analysis.

Since ionizing radiation triggers a cascade of molecular events that ultimately leads to apoptosis, we evaluated the activation of two major proteins of the apoptotic signalling cascade, caspase-3 and poly (ADP-ribose) polymerase (PARP). Our results demonstrated that ionizing radiation enhanced cleaved PARP and cleaved caspase-3 levels in both RKO and SW1463 cells, either cultured alone or in combination with macrophages (Fig 2). Interestingly, in comparison with mono-cultures, the presence of macrophages enhanced both PARP and caspase-3 cleavage in RKO cells, while reduced both proteins cleavage in SW1463 cells, upon radiation exposure (Fig 2).

Notably, the presence of macrophages *per se* (without radiation exposure) decreased cleaved PARP and cleaved caspase-3 expression levels in SW1463 cells, while an increase was verified for RKO cells. These results suggest that macrophages may direct cancer cell response to ionizing radiation, particularly by sensitizing RKO cells and promoting the radioresistance of SW1463 cells to radiation-induced cell death.

In order to understand SW1463 cancer cell enhanced radioresistance in the presence of macrophages, we first evaluated whether it could be attributed to a reduction of ionizing radiation-induced DNA damage signalling. Therefore, the expression and phosphorylation status of the Checkpoint kinase 2 (Chk2), a protein involved in the propagation of DNA damage signals [27], and the phosphorylation of H2AX (Ser139), a DNA double-strand marker, were evaluated 6 h after irradiation (S1 Fig). Our results demonstrated that both Chk2 and H2AX phosphorylation increased in SW1463 irradiated alone or in co-culture, when compared to the respective non-irradiated controls, indicating, as expected, higher DNA damage levels in irradiated cells. However, no major alterations were found between Chk2 phosphorylation levels in SW1463 cells cultured alone or in combination with macrophages, suggesting that macrophages may increase SW1463 radioresistance through alternative mechanisms rather than DNA damage reduction, which requires further investigation.

### Increased expression of metabolism-related genes upon co-culture with macrophages may explain the reduced SW1463 apoptotic signaling after co-culture irradiation

**Fig 3:**
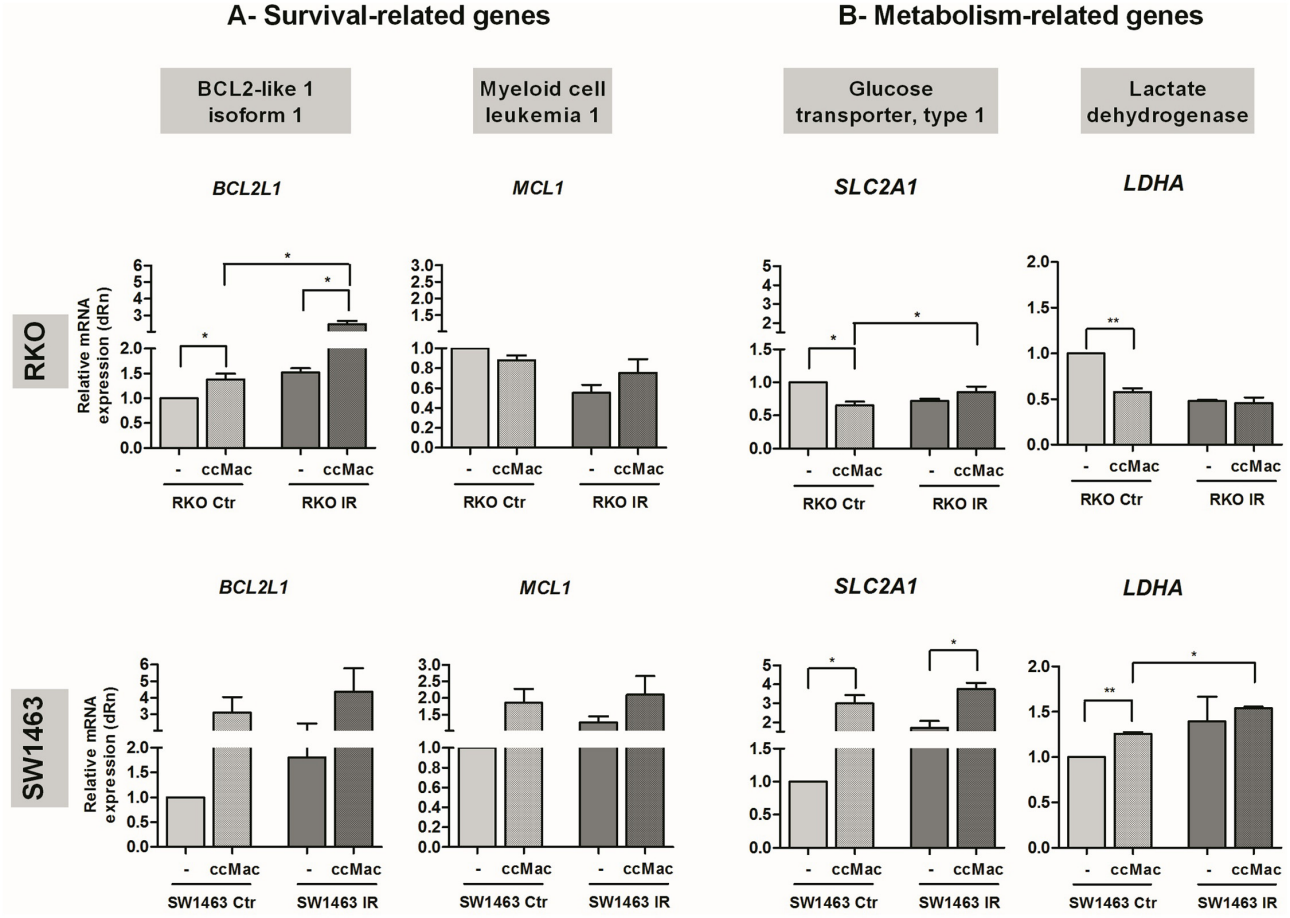
Expression of survival- and metabolism-related targets in RKO and SW1463 cancer cells. Both RKO and SW1463 cancer cells were cultured alone (-) or in the presence of macrophages (ccMac), with (IR, 5 x 2 Gy) or without (Ctr) radiation exposure. The mRNA expression levels of **A)** two survival-related genes, *BCL2L1* and *MCL1*, which encode the anti-apoptotic proteins Bcl-xL and Mcl-1, respectively, and **B)** of two metabolism-related genes, *SLC2A1* and *LDHA*, which encode the glucose transporter type 1 and the lactate dehydrogenase, respectively, were evaluated in cancer cells, 6 h after irradiation. Graphs result from the relative mRNA quantification in cancer cells cultured with macrophages from distinct donors (*n* = 4 per each cell line), evaluated in four independent experiments. * *P* < 0.05, ** *P* < 0.01.

In order to search for possible mechanistic explanations, we focused on two additional targets involved in anti-apoptotic response, Bcl-xL and Mcl-1, encoded by *BCL2L1* and *MCL1*, respectively (Fig 3A). Results demonstrate that upon co-culture with macrophages, RKO cells increased *BCL2L1* expression, without alterations in *MCL1*, independently of ionizing radiation treatment. In SW1463 cells, macrophages tend to increase the expression levels of both *BCL2L1* and *MCL1* upon irradiation, although without achieving statistical significance.

Since mitochondrial dysfunction after radiation exposure may compromise energy supply [28] and cell function [29], we focused on the expression of two metabolism-related genes, *SLC2A1* and *LDHA* (Fig 3B), which frequent overexpression by cancer cells contribute to increased glycolysis [30]. *SLC2A1* encodes the glucose transporter 1 (GLUT1), a membrane-bound protein responsible for glucose uptake, while *LDHA* encodes the lactate dehydrogenase A, an enzyme responsible for the conversion of pyruvate to lactate. Notably, macrophages decreased RKO cells *SLC2A1* and *LDHA* expression, but only in the absence of ionizing radiation. Contrarily, macrophages enhanced SW1463 *SLC2A1* expression in both irradiated and non-irradiated conditions and *LDHA* without radiation exposure. Additionally, an increase of *LDHA* expression in irradiated versus non-irradiated SW1463 co-cultures, but not in RKO co-cultures, was found, suggesting that SW1463 cells exhibit an adaptive metabolic response and a more radiation resistant profile, as indicated by the literature [25].

Overall, our results revealed that for RKO cells, macrophages overall effect leads to enhanced apoptosis, despite the increased mRNA expression of the pro-survival *BCL2L1*. For SW1463, macrophages contribute to decrease apoptosis, which may be achieved through metabolic alterations induced by enhanced glucose transporter expression, which was also validated at the protein level (S2 Fig).

### Colorectal cancer cells promote a macrophage pro-inflammatory phenotype, with or without radiation exposure

Besides interfering with survival- and metabolism-related targets in cancer cells, the presence of macrophages also modulated the expression of macrophage-stimulating factor 1 (*CSF1*), an important molecule for macrophage recruitment. Notably, in response to radiation, the presence of macrophages induced upregulation of *CSF1* in RKO, but not in SW1463 cells (S3 Fig). This result led us to investigate whether RKO and SW1463 cancer cells could differently modulate macrophage response to radiation.

**Fig 4:**
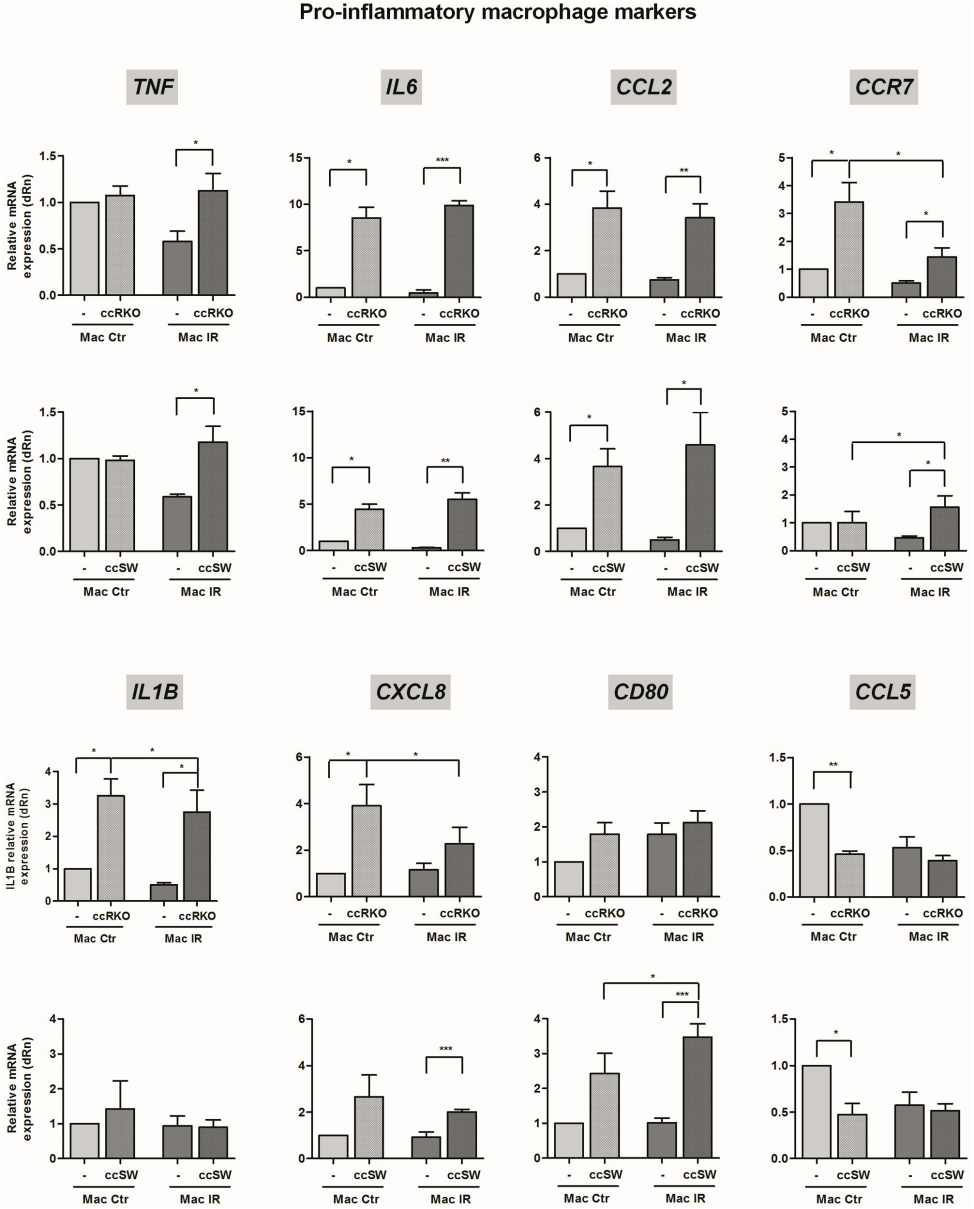
The mRNA expression of pro-inflammatory markers in irradiated macrophages, cultured with RKO or SW1463 cells. Macrophages were cultured alone (-) or in the presence of RKO or SW1463 cancer cells (ccRKO or ccSW), with (IR, 5 x 2 Gy) or without (Ctr) radiation exposure. The mRNA expression of a panel of pro-inflammatory macrophage markers (*TNF*, *IL6*, *CCL2*, CCR7, *IL1B*, *CXCL8*, *CD80* and *CCL5*) was evaluated 6 h after irradiation. Graphs result from the relative mRNA quantification in macrophages cultured with RKO or SW1463 (*n* = 4 per each cell line), evaluated in four independent experiments. For simplicity, SW1463 cells were indicated as “SW”. * *P* < 0.05, ** *P* < 0.01, *** *P* < 0.001.

**Fig 5:**
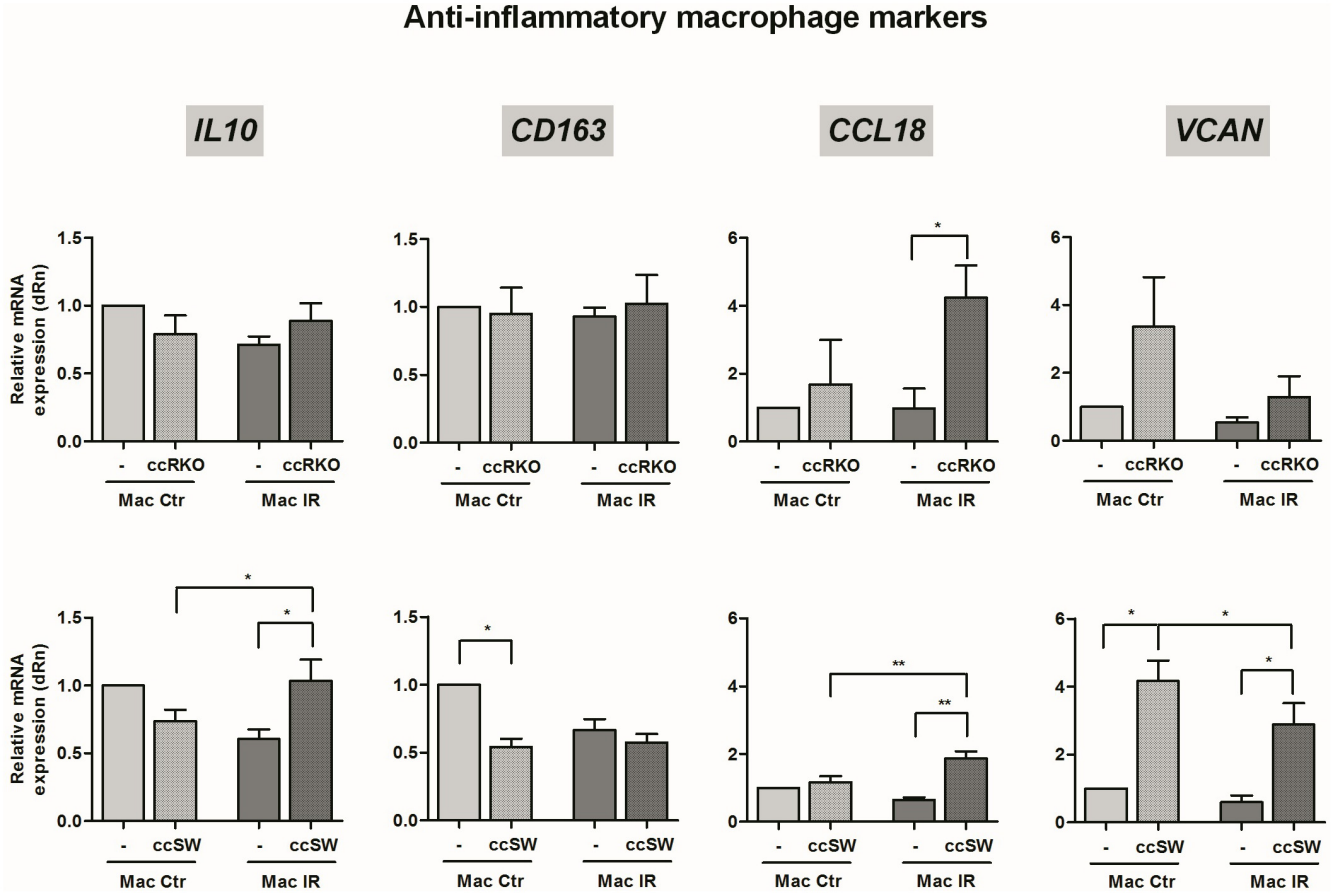
The mRNA expression of anti-inflammatory markers in irradiated macrophages, cultured with RKO or SW1463 cells. Macrophages were cultured alone (-) or in the presence of RKO or SW1463 cancer cells (ccRKO or ccSW), with (IR, 5 x 2 Gy) or without (Ctr) radiation exposure. The mRNA expression of a panel of anti-inflammatory macrophage markers (*IL10*, *CD163*, *CCL18* and *VCAN*) was evaluated 6 h after irradiation. Graphs result from the relative mRNA quantification in macrophages cultured with RKO or SW1463 (*n* = 4 per each cell line), evaluated in four independent experiments. For simplicity, SW1463 cells were indicated as “SW”. * *P* < 0.05, ** *P* < 0.01, *** *P* < 0.001.

To address this question, we compared the inflammatory profile of macrophages cultured in the presence of each of the colorectal cancer cells with the one of macrophages monocultures, 6 h after radiation exposure (third versus fourth column of each graph, Figs 4 and 5). The macrophage inflammatory profile was assessed by mRNA expression analysis of a panel of pro-inflammatory (*CD80*, *CCL2*, *CXCL8*, *TNF*, *CCR7*, *IL6*, *IL1B* and *CCL5*) and anti-inflammatory (*CD163*, *IL10*, *CCL18* and *VCAN*) genes.

Our results demonstrated that in the presence of ionizing radiation either RKO or SW1463 cancer cells increased mRNA expression levels of the macrophage pro-inflammatory markers *TNF*, *IL6*, *CCL2 and CCR7* (Fig 4) as well as of the anti-inflammatory marker *CCL18* (Fig 5). Despite these molecular similarities, RKO, but not SW1463 cells, enhanced the expression of the macrophage pro-inflammatory marker *IL1B*. Additionally, SW1463, but not RKO cells, increased the expression of *CXCL8* and *CD80* pro-inflammatory (Fig 4), and of *VCAN* and *IL10* anti-inflammatory markers (Fig 5).

Particularly, we observed that, in the absence of ionizing radiation both colorectal cancer cells had already the ability to modulate macrophage inflammatory profile (first versus second column of each graph, Figs 4 and 5). Consistently, in the presence of either RKO or SW1463 cells, macrophages exhibited increased expression of *IL6 and CCL2*, while decreased the expression of *CCL5* pro-inflammatory markers. Although *CXCL8* and *VCAN* tend to increase in macrophages cultured with both cancer cells, statistical significance was only achieved for the anti-inflammatory marker *VCAN* in co-cultures with SW1463 and for the pro-inflammatory marker *CXCL8* marker in co-cultures with RKO cells. In addition, RKO also induced a significant increase of *IL1B* and *CCR7* pro-inflammatory markers, while SW1463 cells significantly reduced the expression of the anti-inflammatory *CD163* receptor. Altogether, these data suggest that both RKO and SW1463 colorectal cancer cells promoted, even in the absence of ionizing radiation, a macrophage pro-inflammatory phenotype.

To distinguish the molecular alterations dependent on irradiation from those induced by the presence of colorectal cancer cells, the mRNA expression levels of macrophages from irradiated co-cultures were compared with those from non-irradiated co-cultures (second versus fourth column of each graph, Figs 4 and 5). Most strikingly, our results evidenced that ionizing radiation significantly reduced the expression of *CCR7* pro-inflammatory receptor on macrophages co-cultured with RKO, while enhanced it on macrophages co-cultured with SW1463 cells (Fig 4). Additionally, for co-cultures established in the presence of RKO cells, radiation also decreased significantly the expression of the pro-inflammatory chemokine *CXCL8* (Fig 4). However, for co-cultures established in the presence of SW1463 cells, ionizing radiation increased significantly the expression of the pro-inflammatory *CD80* receptor and of the *CCL18* cytokine (Fig 4) and of the anti-inflammatory *IL10* cytokine, while reduced the expression of the anti-inflammatory extracellular matrix *VCAN* (Fig 5). No major alterations were found between the levels of the pro-inflammatory *TNF*, *IL6*, *CCL2* and *CCL5* and of the anti-inflammatory *CD163* in macrophages cultured in the presence of cancer cells, with or without radiation exposure (Figs 4 and 5).

### Conditioned medium from irradiated macrophage-RKO co-culture increased migration and invasion of non-irradiated RKO cells

**Fig 6:**
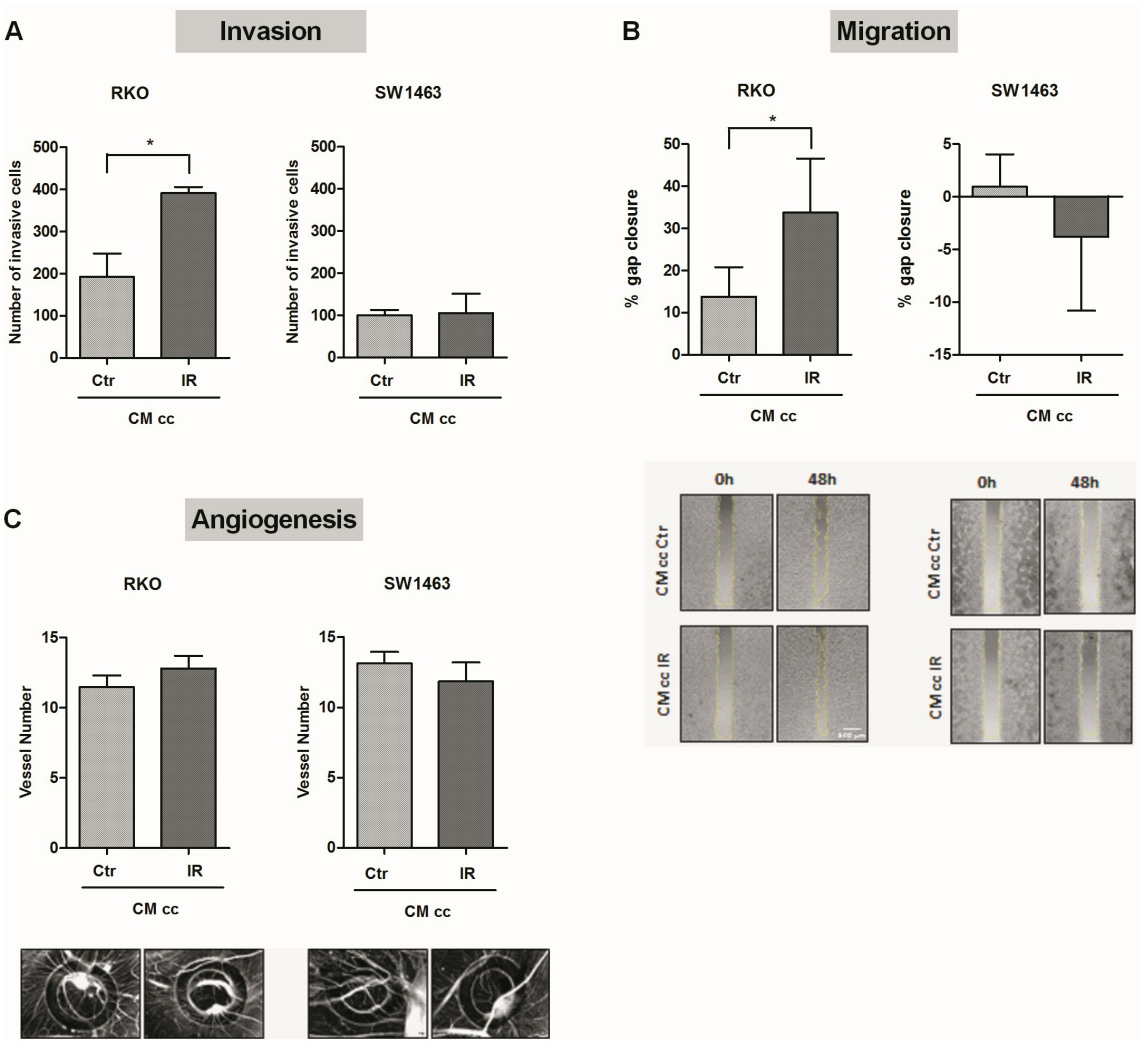
Effect of conditioned medium from irradiated co-cultures in the invasion, migration and angiogenesis of non-irradiated cells. The ability of conditioned medium (CM), from irradiated (IR, 5 x 2 Gy) and non-irradiated (Ctr) co-cultures (cc) of macrophages with RKO or SW1463, to promote cancer cell migration and invasion (*n* = 4 per each cell line), as well as to induce angiogenesis, was evaluated. **A)** Non-irradiated RKO and SW1463 cells were seeded on Matrigel-based transwells and stimulated with CM from irradiated and non-irradiated co-cultures. After 24 h, the number of invasive cells was counted. **B)** Both non-irradiated RKO or SW1463 were plated until confluence on both chambers of Ibidi culture-inserts for migration assay. After wound formation, cancer cells were stimulated with CM from irradiated and non-irradiated co-cultures and migrated area was quantified upon 48 h. **C)** Concentrated CM from irradiated or non-irradiated co-cultures was inoculated in rings, on the top of the chick embryo chorioallantoic membrane (CAM), for 72 h. Analysis of CM-induced angiogenesis was performed through quantification of the number of new vessels in control and experimental conditions.

To evaluate how irradiated co-cultures affected the activity of non-irradiated cells, the ability of conditioned medium (CM) from irradiated and non-irradiated co-cultures to interfere with some hallmarks of cancer, namely invasion, migration and angiogenesis, was investigated (Fig 6). We have previously described that invasion, the hallmark of cancer that involves the ability of cancer cells to cross the basement membrane and migrate through the nearby tissues, is also mediated by factors released by tumour-associated cells, as macrophages [1, 26]. Considering this, the invasion ability of non-irradiated cancer cells was evaluated in the presence of CM from either irradiated or non-irradiated co-cultures, using transwell inserts with a porous membrane coated with Matrigel, mimicking the basement membrane matrix (Fig 6A). Our results revealed that CM from irradiated co-cultures significantly increased the invasion of non-irradiated RKO (*P* < 0.05), in comparison with CM from non-irradiated co-cultures. However, no major alterations were observed in the invasion ability of SW1463 cells stimulated with CM from irradiated and non-irradiated co-cultures. For the analysis of cancer cell migration, non-irradiated RKO or SW1463 cancer cells were grown until confluence in a two chamber well and then stimulated with CM from irradiated or non-irradiated macrophages-cancer cell co-cultures (Fig 6B). After wound formation, RKO or SW1463 were able to migrate from both chambers towards the centre. Results demonstrated that after 48 h, CM from irradiated co-cultures stimulated RKO migration (*P* < 0.05), filling the empty area of the wound more efficiently than RKO stimulated with CM from non-irradiated co-cultures. However, no migration ability was observed for SW1463 in the presence of CM of either irradiated or non-irradiated co-cultures.

Since macrophages and cancer cells are able to induce angiogenesis through the secretion of several pro-angiogenic growth factors and cytokines/chemokines [1], the angiogenic potential of CM from irradiated and non-irradiated co-cultures was evaluated using the chick chorioallantoic membrane (CAM) model (Fig 6C). Notably, CM from irradiated co-cultures of macrophages with either RKO or SW1463 cancer cells had a similar angiogenic response as CM from non-irradiated co-cultures.

## Discussion

Our results demonstrated that macrophages enhance the cancer cell intrinsic response to radiation, this is, they promote RKO radiation sensitivity, while enhance SW1463 cells radioresistance. This effect could be attributed either to a different ability of the distinct cell lines to modulate macrophage phenotype, to a differential response to the presence of macrophages upon irradiation, or most probably, to a combination of both.

The communication of macrophages with cancer cells, and how both populations influence each other, has been explored by several studies, at both cellular and molecular levels. Most of those *in vitro* studies rely on two main strategies, direct [31, 32] or indirect [33–35] macrophage-cancer cell co-cultures or, alternatively, stimulation of one population with the supernatant of the other [36, 32, 37]. The preferential methodological approach seems to be indirect macrophage-cancer cell co-cultures, as this constitutes a simple and practical *in vitro* model. These studies also frequently use macrophage-like cells differentiated from THP-1 human monocytes [33, 35, 36], upon stimulation with phorbol myristate acetate (PMA), although these are not completely representative of primary human macrophages [38]. Although the knowledge about macrophage-cancer cell communication has improved in the last years, there is scarce information on how ionizing radiation may modulate macrophage inflammatory profile and how the molecular crosstalk established between macrophages and cancer cells affects the radiation response of both populations.

To address these questions, we established indirect co-cultures between primary human macrophage cultures and colorectal cancer cells (CRCs), using two cell lines with different radiation sensitivities [25, 24]. Briefly, radiosensitive RKO cells express mutated *ATM* [39], similarly to patients with a genetic disorder characterized by hypersensitivity to ionizing radiation [40]. On the other hand, SW1463 cells express mutated *TP53* and *KRAS* [39], which may delay apoptosis [41], enhance repair of DNA double-strand breaks [42] or induce metabolic reprogramming [43, 42], as a way of protecting cells from radiation-induced damage.

Besides mutations, that intrinsically determine cancer cell response to radiation, other events, with which macrophages may interfere, can also modulate it. Our data demonstrated that in the presence of macrophages, radioresistant SW1463 cells increased *SLC2A1*, *MCL1*, and *BCL2L1* to levels very similar to those exhibited without irradiation, suggesting that the presence of macrophages *per se* may induce some protection against ionizing radiation. In general, cancer cells with acquired radioresistance exhibit higher expression of the glucose transporter GLUT-1 (encoded by *SLC2A1*) and enhanced lactate production levels than parental cells, which are maintained or increased upon radiation exposure [44]. Accordingly, some authors reported that although transient, the higher glucose uptake seems to occur concomitantly with increased *SLC2A1* mRNA levels [45]. Additionally, upregulation of *MCL1* mRNA expression upon ionizing radiation exposure was reported as an early response to cytotoxic stress, reaching its peak at 4 h after a single 20 Gy dose, backing to basal levels 24 h after irradiation [46]. It was hypothesized that the maintenance of high *MCL1* levels or their increase upon irradiation, associated with enhanced short-term cell viability, could allow repair of radiation-induced DNA damage and suppression of apoptotic response, even in the presence of high levels of active caspase-3 [47, 46], [48]. Accordingly, Mcl-1 targeting or depletion may sensitize cancer cells to radiation-induced apoptosis [49]. Overall, it seems that Mcl-1 acts as an integrative signal, mediating the opposite actions of pro-survival and pro-apoptotic signalling [50], thereby being an attractive target for modulation of cell radioresistance. On its turn, Bcl-xL targeting was reported to reduce CRCs survival upon ionizing radiation exposure by highly increasing their apoptotic rate [51]. In addition to macrophage induced alterations in cancer cell´s metabolic and pro-survival targets, the modulation of macrophage polarization profile by cancer cells may also help to explain why both RKO and SW1463 cancer cell lines responded differently to radiation in the presence of macrophages. In that respect, the increased expression, in irradiated macrophages upon co-culture with RKO cells, of the pro-inflammatory *IL1B*, a crucial molecule for macrophage-mediated tumouricidal activity [52], may support the macrophage-increased RKO radiosensitivity. On the other hand, increased levels of anti-inflammatory *IL10* in irradiated macrophages upon co-culture with SW1463 cells may support survival and consequent macrophage-promoted SW1463 radioresistance, since high *IL10* levels in tumour-associated macrophages may play a role in cancer progression [53] and resistance to therapy [54]. Additionally, we may also speculate that the simultaneous increase, in irradiated macrophages upon co-culture with SW1463 cells, of pro-inflammatory *CD80*, a co-stimulatory molecule involved in antigen presentation, could be an attempt of macrophages to overcome their apparent lack of tumouricidal activity on SW1463 cells.

Although our main goal was to explore the importance of macrophage-cancer cell communication in response to radiation, it became relevant also to investigate that crosstalk without radiation exposure. In that respect, several studies start to point some common conclusions, such as both macrophages and cancer cells are mutually affected when in co-culture, and also that macrophages may develop a mixed M1/M2 phenotype, whose exact profile depends on the selected cancer cell line [36]. For instance, supernatants of two CRC lines with different pathological status, HT-29 (Dukes’ B stage, meaning invasion into the muscle layer of the bowel) and Colo205 (Dukes’ D stage, meaning advanced CRC), induced a more pro-inflammatory (M1-like) or anti-inflammatory (M2-like) phenotype in PMA-treated THP-1 cells, respectively [36]. Accordingly, and using a similar *in vitro* system to the one we used, Hollmén and colleagues co-cultured, for 5 days, human monocytes with two breast cancer lines. Their data suggested the induction of a pro-inflammatory macrophage phenotype upon co-culture with T47D cells, which are less invasive and respond well to anti-hormonal therapy, and of an anti-inflammatory phenotype upon co-culture with MDA-MB-231 cells, which are highly invasive cancer cells with lack of effective treatment [34, 55]. Altogether these studies support our results, demonstrating a mutual influence between macrophages and cancer cells, which, although under non-irradiation conditions, may be crucial for cell response to radiation. However, we cannot exclude that these interactions may be different *in vivo*, where macrophage-cancer cell crosstalk is subjected to the influence of the other cells from the tumour microenvironment.

Finally, by studying how irradiated cells affect those that have not been directly exposed to ionizing radiation, a phenomenon termed as radiation-induced bystander effect [56], we demonstrate that, contrary to non-irradiated SW1463, RKO cells become more invasive and migrate more in the presence of irradiated macrophage-cancer cell co-culture-released signals. This suggests that, although the enhancement of SW1463 radioresistance by macrophages constitutes a motif of concern, attention should also be paid to the non-targeted effects of radiotherapy, particularly those mediated by radiosensitive cells, like RKO.

## Conclusions

**Fig 7:**
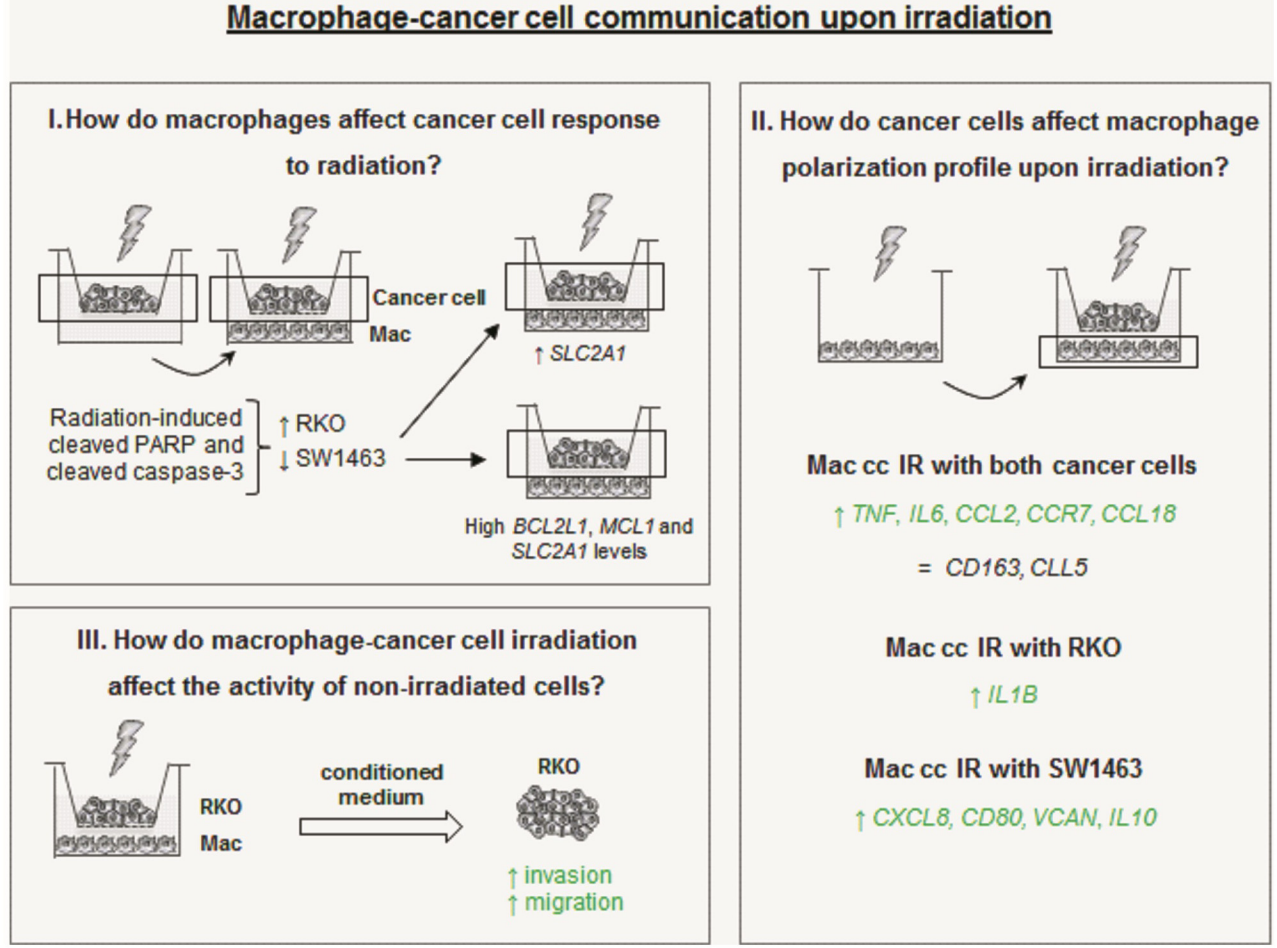
Intricate macrophage-cancer cell communication upon irradiation. **I)** In the presence of macrophages, radiosensitive RKO cell increased cleaved PARP and cleaved caspase-3 expression in response to radiation, while radioresistant SW1463 reduced the expression of these targets. The high *BCL2L1*, *MCL1* and *SLC2A1* levels observed in SW1463 cells upon co-culture with macrophages, together with the increased expression of *BCL2L1* in irradiated SW1463 upon co-culture with macrophages, may contribute to macrophage-induced SW1463 enhanced radioresistance. **II)** In the presence of either RKO or SW14363 cancer cells, irradiated macrophages exhibit higher levels of pro-inflammatory *TNF*, *IL6*, *CCL2* and *CCR7*, but also of anti-inflammatory *CCL18*, being differences in other targets dependent on the nature of the cancer cells with which macrophages were cultured. Thus, in the presence of RKO cells irradiated macrophages exhibit an increase of pro-inflammatory *IL1B*, while SW1463 cells promote higher pro-inflammatory *CXCL8* and *CD80*, but also anti-inflammatory *VCAN* and *IL10* levels. **III)** Conditioned medium (CM) from macrophage-RKO irradiated co-culture induced increased invasion and migration of non-irradiated RKO cells. Abbreviations: cc–co-cultures; IR–irradiated.

Altogether this data reinforce our previous results [23], demonstrating that ionizing radiation modulates macrophage profile towards a more pro-inflammatory one and that this is also the case when macrophages are co-cultured with colorectal cancer cells. Furthermore, the molecular crosstalk established between macrophages and cancer cells seems to modulate the response of the latest to ionizing radiation exposure. Remarkably, the present *in vitro* approach demonstrated that macrophages enhance the cancer cell intrinsic response to radiation, promoting RKO radiation sensitivity, while enhancing SW1463 cells radioresistance. This will depend on the intrinsic nature of each cancer cell, how it responds to macrophage presence, as well as on their modulation of macrophage polarization profile (Fig 7). Overall, a better understanding of the mechanisms responsible for cancer cell radioresistance will contribute to the discovery of potential cellular and molecular targets to improve radiotherapy efficacy [57].

## Supporting Information

S1 Fig Evaluation of Chk2 and H2AX (Ser139) phosphorylation levels in SW1463 cancer cells. SW1463 cells were irradiated (IR, 5 x 2 Gy) alone (-) or in co-culture with macrophages (ccMac). Chk2 phosporylation (Thr 387), total Chk2 and phosphorylated H2AX (Ser139, ɣH2AX) were evaluated, by western blot analysis, 6 h after irradiation.
(TIF)

S2 Fig Evaluation in GLUT1 expression in SW1463 cancer cells. SW1463 cancer cells were cultured alone (-) or in the presence of macrophages (ccMac), with (IR, 5 x 2 Gy) or without (Ctr) radiation exposure. GLUT1 protein expression levels were evaluated in cancer cells, 6 h after irradiation.
(TIF)

S3 Fig Evaluation of *CSF1* mRNA expression in RKO and SW1463 cancer cells. Both RKO and SW1463 cancer cells were cultured alone (-) or in the presence of macrophages (ccMac), with (IR, 5 x 2 Gy) or without (Ctr) radiation exposure. CSF1 mRNA expression levels were evaluated in cancer cells, 6 h after irradiation. Graphs result from the relative mRNA quantification in cancer cells cultured with macrophages from distinct donors (*n* = 4 per each cell line), evaluated in four independent experiments. * *P* < 0.05.
(TIF)
