# Supplementary figures and images for: Intricate Macrophage-Colorectal Cancer Cell Communication in Response to Radiation
Source: PLoS One. 2016 Aug 11;11(8):e0160891. doi: 10.1371/journal.pone.0160891 (PMC4981353; doi:10.1371/journal.pone.0160891)

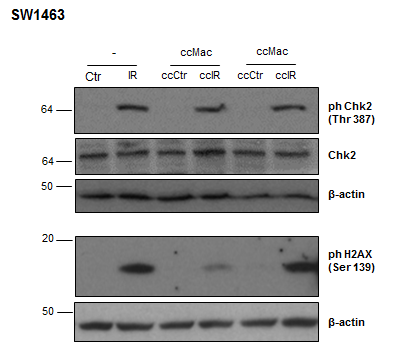

Supplement: S1 Fig — SW1463 cells were irradiated (IR, 5 x 2 Gy) alone (-) or in co-culture with macrophages (ccMac). Chk2 phosporylation (Thr 387), total Chk2 and phosphorylated H2AX (Ser139, ɣH2AX) were evaluated, by western blot analysis, 6 h after irradiation. (TIF) [file pone.0160891.s001.tif]

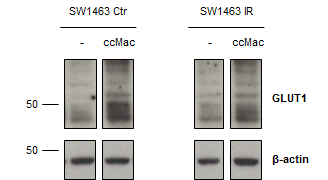

Supplement: S2 Fig — SW1463 cancer cells were cultured alone (-) or in the presence of macrophages (ccMac), with (IR, 5 x 2 Gy) or without (Ctr) radiation exposure. GLUT1 protein expression levels were evaluated in cancer cells, 6 h after irradiation. (TIF) [file pone.0160891.s002.tif]

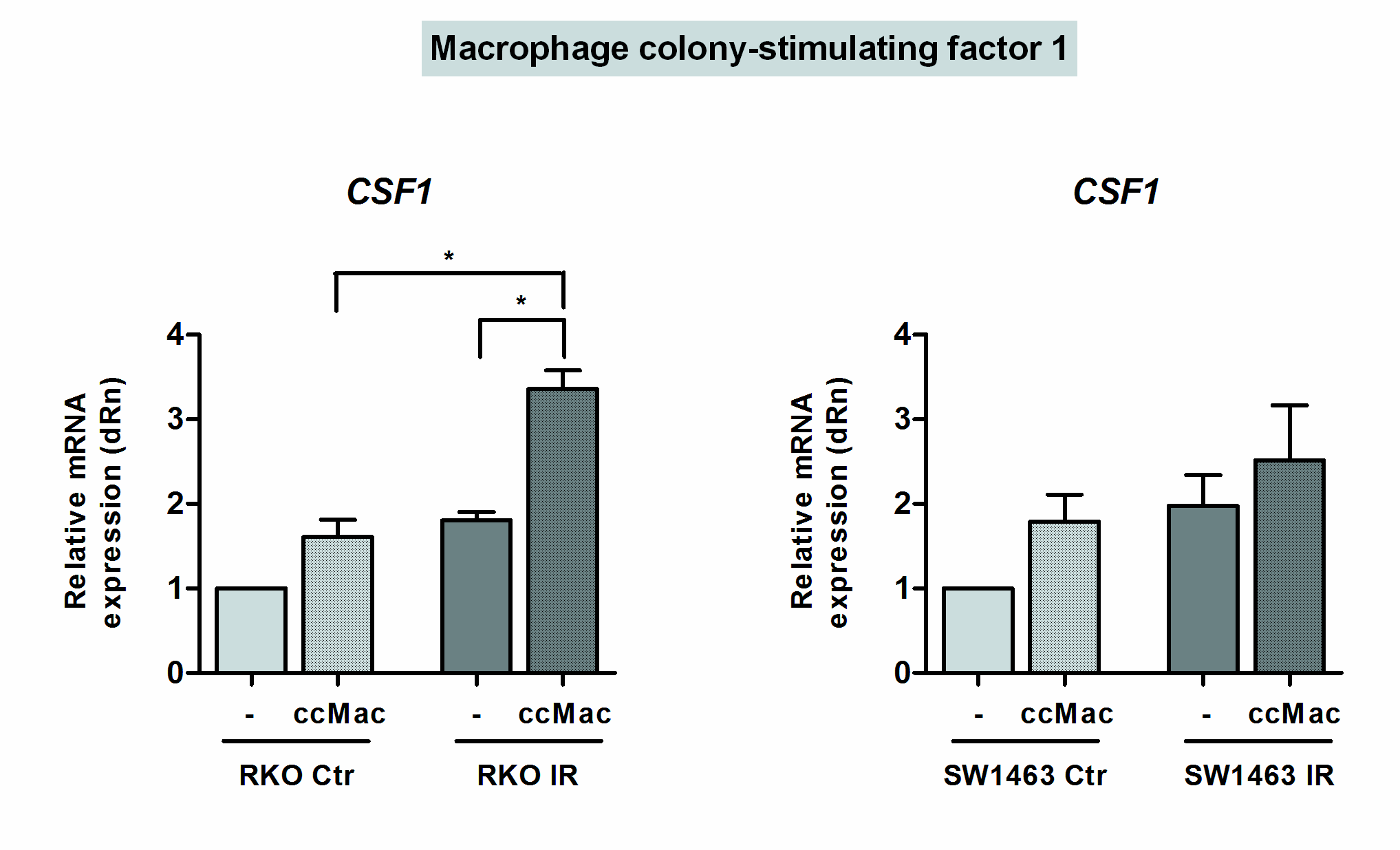

Supplement: S3 Fig — Both RKO and SW1463 cancer cells were cultured alone (-) or in the presence of macrophages (ccMac), with (IR, 5 x 2 Gy) or without (Ctr) radiation exposure. CSF1 mRNA expression levels were evaluated in cancer cells, 6 h after irradiation. Graphs result from the relative mRNA quantification in cancer cells cultured with macrophages from distinct donors (n = 4 per each cell line), evaluated in four independent experiments. * P < 0.05. (TIF) [file pone.0160891.s003.tif]
